# Elimination of congenital syphilis: a nationwide register-based study, Denmark, 2010 to 2024

**DOI:** 10.2807/1560-7917.ES.2026.31.35.2600031

**Published:** 2026-09-03

**Authors:** Ulrik Lausten-Thomsen, Steen Hoffmann, Paula Louise Hedley, Lene Holm Harritshøj, Jan Gorm Lisby, Marie Bækvad-Hansen, Michael Christiansen, Maria Wessman

**Affiliations:** 1 Department of Neonatology, Copenhagen University Hospital Rigshospitalet, Copenhagen, Denmark; 2 Department for Congenital Disorders, Statens Serum Institut, Copenhagen, Denmark; 3 Department of Bacteria, Parasites and Fungi, Statens Serum Institut, Copenhagen, Denmark; 4 Department of Epidemiology, College of Public Health, The University of Iowa, Iowa City, Iowa, United States; 5 Department of Clinical Immunology, Copenhagen University Hospital Rigshospitalet, Copenhagen, Denmark; 6 Department of Clinical Medicine, Faculty of Health and Clinical Sciences, Copenhagen University, Copenhagen, Denmark; 7 Department of Clinical Microbiology, Copenhagen University Hospital Hvidovre, Copenhagen, Denmark; 8 Department of Biomedical Sciences, University of Copenhagen, Copenhagen, Denmark; 9 Department of Infectious Disease Epidemiology and Prevention, Statens Serum Institut, Copenhagen, Denmark

**Keywords:** mass screening, pregnancy, prenatal care, syphilis, syphilis, congenital

## Abstract

**BACKGROUND:**

Congenital syphilis is a potentially serious, but preventable infection. Several European countries have recently reported increasing trends.

**AIM:**

This study assessed national incidence and temporal trends of congenital syphilis and prevalence of pregnancies with active syphilis in Denmark between 2010 and 2024.

**METHODS:**

Using nationwide individual-level data linked through civil registration numbers across Danish health and population registers, we identified all maternal and congenital syphilis cases among live-born children born in Denmark from 2010 to 2024. We calculated annual incidence per 10,000 live births and analysed temporal trends and annual percent change (APC) with 95% confidence intervals (CI), using Poisson or negative binomial regression models.

**RESULTS:**

Five infants with confirmed congenital syphilis were recorded among 894,345 live births, corresponding to an incidence of 0.6 per 100,000 (95% CI: 0.2–1.4). No infants with congenital syphilis were identified after 2016. For each infant, a plausible explanation for screening failure was identified: two women arrived in Denmark late in pregnancy, two were infected late in pregnancy. None of these events reflected failure of the first-trimester screening algorithm itself. One event was due to a reporting irregularity. Poisson regression showed a decreasing trend (APC = −21.7%; 95% CI: −43.4 to −1.0). Active syphilis infection occurred in 172 pregnancies, amounting to 20.0 per 100,000 live births (95% CI: 17.2–23.2) with no temporal changes.

**CONCLUSIONS:**

Congenital syphilis is now eliminated in Denmark. The incidence of pregnancies with active syphilis infection remained stable despite increasing syphilis prevalence in the general population, confirming the continued effectiveness of the Danish national antenatal syphilis screening programme.

**Key public health message**

**What did you want to address in this study and why?**

Congenital syphilis occurs when syphilis is transmitted from an infected, pregnant woman to her fetus. Without timely treatment, it can cause stillbirth, preterm birth, neonatal death and serious long-term complications. Because increasing congenital syphilis has been reported in several European countries, we examined congenital syphilis and syphilis-affected pregnancies in Denmark from 2010 to 2024 to see if there is a similar trend.

**What have we learnt from this study?** Congenital syphilis has been eliminated in Denmark, with no cases identified since 2016.

The five cases identified between 2010 and 2016 were explained by late maternal infection or late immigration. Maternal syphilis prevalence remained stable over time, although the prevalence of the infection in the general population was rising.

**What are the implications of your findings for public health?**

Universal first-trimester antenatal screening effectively prevents congenital syphilis. Early access to prenatal care remains critical, especially for mobile and migrant populations. Sustained surveillance is essential to maintain elimination.

## Introduction

Congenital syphilis results from vertical transmission of *Treponema pallidum* from a pregnant woman to the fetus or newborn and remains a serious, yet entirely preventable, cause of adverse birth outcomes worldwide. Untreated maternal infection can lead to stillbirth, preterm delivery, neonatal death and severe neonatal morbidity including bone deformities, deafness and neurological sequelae [1,2]. Since antibiotics remain highly effective against both maternal and fetal infection [3], elimination of vertical transmission of syphilis has long been recognised as an achievable global goal [4,5].

In 2007, the World Health Organization (WHO) launched the initiative for the global elimination of congenital syphilis, emphasising universal screening in pregnancy, timely treatment and strengthened surveillance systems [6]. However, progress has been uneven, and several regions have reported increases in congenital syphilis. In the United States, incidence rose more than 10-fold between 2012 and 2022 [7], and upward trends have also been reported in parts of Europe, including Portugal, the United Kingdom, and some countries in eastern Europe [8-10]. These resurgences appear to be driven by rising syphilis prevalence among women of reproductive age and missed opportunities for screening or treatment during pregnancy [11].

Antenatal screening and prompt antibiotic therapy are highly effective in preventing congenital syphilis. Most European countries, including Denmark, have implemented nationwide first-trimester screening programmes using treponemal and non-treponemal assays [12]. When screening coverage is high and treatment is adequate, congenital syphilis becomes exceedingly rare, and any new occurrence serves as a sentinel event signalling potential gaps in prevention or healthcare access [4,6,7].

In Denmark, syphilis screening during pregnancy was implemented in the late 1980s based on individual risk assessment, and became universal in 2010 with coverage exceeding 99% of live births [13]. Owing to this long-standing programme, congenital syphilis has become increasingly rare. Yet given the severity of outcomes and the existence of an evidence-based prevention strategy, continuous surveillance of congenital syphilis is essential, particularly given increasing syphilis notifications in other European countries. Up-to-date Danish national data are necessary to assess whether maternal and congenital syphilis remain under control.

The aim of this study was therefore to describe nationwide trends of confirmed maternal syphilis during pregnancy and congenital syphilis in Denmark from 2010 to 2024, using register-based data from national health and birth registries.

## Methods

### Data sources and study sample

Universal antenatal syphilis screening was established in Denmark in 2010 and is offered to all pregnant women as part of the comprehensive national free-of-charge prenatal care programme [13]. The programme, coordinated by the Danish Health Authority (*Sundhedsstyrelsen*) and implemented through general practitioners, is carried out by the five national blood banks. Screening is performed at the first antenatal visit, typically in gestational weeks 6–12 and concurrently with blood group typing, contributing to the very high participation rates. It uses a serological algorithm consisting of an initial specific anti-*Treponema pallidum* (anti-TP) enzyme immunoassay performed in one of five regional laboratories. If positive, it is followed by a repeated anti-TP test and a confirmatory non-treponemal test (rapid plasma reagin) in one of three referral laboratories.

Coverage of the antenatal syphilis screening programme in Denmark exceeds 99%, and treatment uptake among seropositive women is close to 100% [13]. Syphilis, including congenital syphilis, is a mandatory notifiable infection in Denmark, and positive laboratory results are automatically reported to the national surveillance system located at Statens Serum Institut [14]. For this study, we linked data from the national surveillance system, the Danish Microbiology Database (MiBa), Danish National Patient Register, Danish Civil Registration System and Danish National Biobank Register. These combined registries enable near-complete ascertainment of both maternal infections and neonatal outcomes.

### Case classification

We included data on all syphilis-positive children below the age of 15 years, live-born in Denmark between 2010 and 2024, and all results from the antenatal syphilis screening during the same time period. For each syphilis-positive child, a qualitative evaluation of confirmatory samples and conditions of suspected timing of infection was done by all the authors using the original data from the mandatory reports including laboratory results and individual-level information. Congenital syphilis was defined as per the European Centre for Disease Prevention and Control [15], based on direct detection of *T. pallidum* in relevant specimens or detection of *T. pallidum*-specific IgM together with a reactive non-treponemal test in the child’s serum. In the same time period, we also included data on all not pregnancy-related new events of syphilis infection from the national surveillance system, including data on year of infection, age, sex and self-reported sexuality.

### Statistics

For each calendar year, we identified (i) the number of syphilis infections detected during pregnancy, (ii) total number of screened pregnancies, (iii) the number of confirmed congenital syphilis cases, and (iv) the total number of live births. Annual incidences of syphilis in pregnancy and congenital syphilis were calculated per 100,000 screened pregnancies and live births for 2010 to 2024, respectively, with exact 95% confidence intervals (CI) estimated assuming a Poisson distribution.

Temporal trends were assessed using Poisson regression, modelling the annual number of cases with the log of live births as an offset. Regression coefficients were exponentiated to obtain incidence rate ratios (IRR), representing the multiplicative change in incidence per calendar year. Annual percent change (APC) was derived from IRRs. Profile-likelihood methods were used to compute 95% CIs for IRRs and APCs. Overdispersion was assessed using the Pearson dispersion statistic; when present, we applied quasi-Poisson or negative binomial models. Quadratic terms were explored to assess potential nonlinear trends and retained only if statistically significant. The level of statistical significance was set at 0.05. All analyses were conducted in R (version 4.3.2; R Foundation for Statistical Computing, Vienna, Austria). Graphs were produced using the *ggplot2* package.

## Results

### Incidence of congenital syphilis

From 2010 to 2024, a total of five cases of congenital syphilis were recorded among 894,345 live births in Denmark, corresponding to an overall incidence of 0.6 per 100,000 live births (95% CI: 0.2–1.4). Cases occurred sporadically (0–1 per year) with none reported since 2016. Poisson regression, adjusting for annual birth counts showed a decreasing temporal trend (β = −0.244; standard error (SE) = 0.136; p = 0.073) corresponding to an average APC of −21.7% (95% CI: −43.4% to −1.0).

For each of the five congenital infections, we identified a plausible identifiable explanation for failure of prevention through the screening programme: two women had recently arrived in Denmark late in pregnancy, two acquired infections late in pregnancy, and in one case a reporting irregularity led to delayed follow-up. Details are summarised in the Table.

**Table:** Congenital syphilis in children born in Denmark, 1 July 2010–31 December 2024 (n = 5)

Patient ID	Year of birth	Age at diagnosis, years	Identified at screening	Clinical signs/symptoms
1	2010	0	No^a^	Unknown
2	2012	0	Yes^b^	No symptoms
3	2013	0	No^c^	Unknown
4	2015	4	No^d^	No symptoms
5	2016	0	Yes^e^	Palmar/plantar desquamation; rhinitis

^a^ The mother was a tourist, not part of the Danish screening programme.

^b^ Positive screening finding overlooked until shortly before birth due to a reporting irregularity.

^c^ Negative screening, the mother is presumed infected in the last part of pregnancy.

^d^ Negative screening, the mother is presumed infected in the last part of pregnancy; this child was identified when the mother tested positive while being pregnant with a younger sibling.

^e^ Found at a late screening as the mother arrived in Denmark 1 week before giving birth.

Three additional children born outside Denmark and arriving in Denmark during early childhood, were identified in national registers with presumed congenital syphilis but were excluded from the analyses, as they were not part of the domestic birth cohort. Another infant was previously misclassified as having congenital syphilis and was corrected during this study’s validation process.

### Antenatal screening results and pregnancies with confirmed active syphilis infection

During the study period, 963,424 pregnant women were tested for syphilis, corresponding to an overall testing rate of 99.1%. The testing rate was > 99.5% in the most recent decade.

Among screened pregnancies, 1,720 women had an initial positive screening test (178.5 per 100,000 tested pregnancies; 95% CI: 170.2–187.2). Of these, 172 (10.0%; 95% CI: 8.6–11.5) were confirmed positive for active infection in this pregnancy after supplementary testing, corresponding to 17.8 pregnancies with confirmed active syphilis infection per 100,000 tested pregnancies (95% CI: 15.3–20.7).

**Figure 1:**
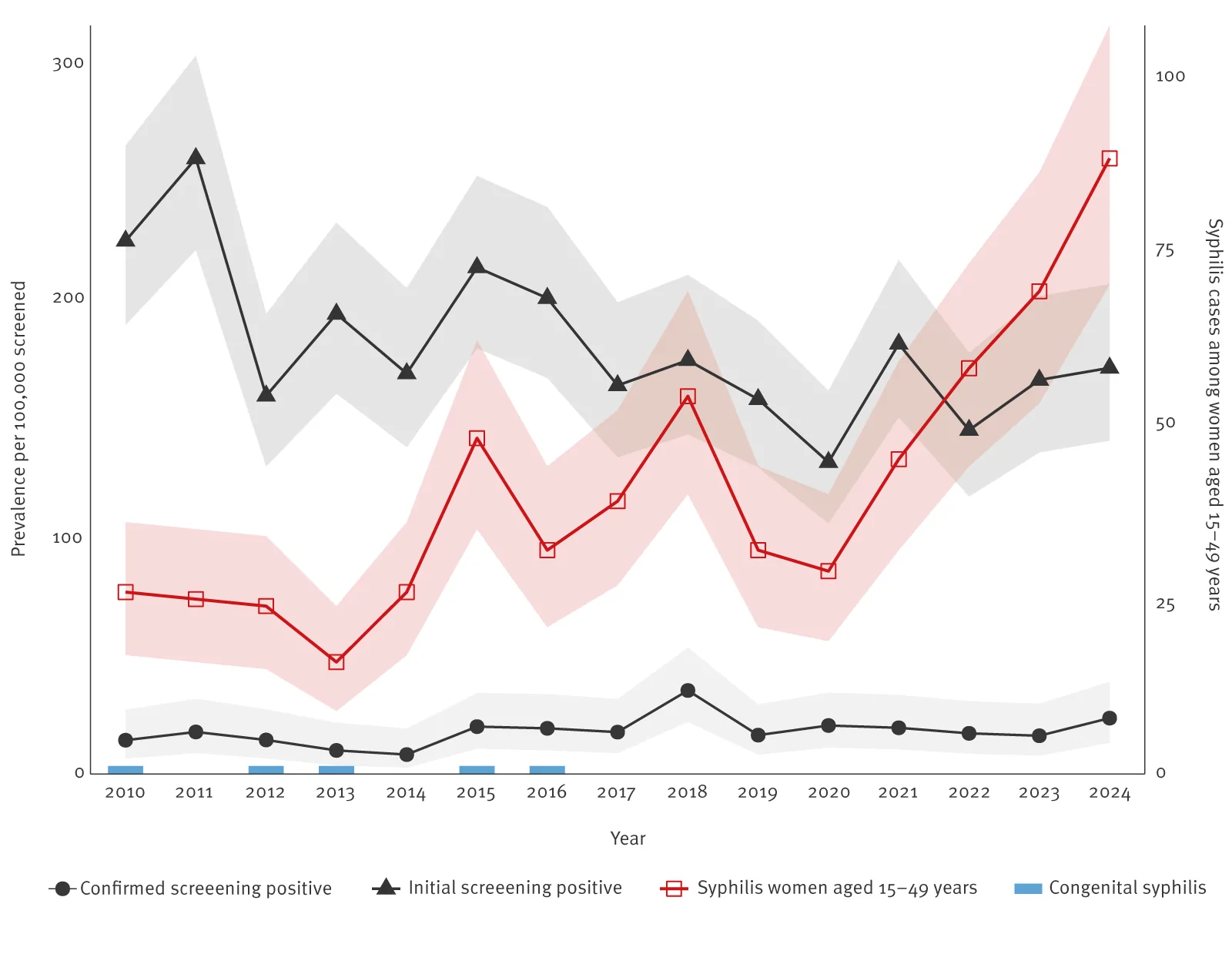
Prevalence per 100,000 of initial positive syphilis screening, pregnancies with confirmed active syphilis, congenital syphilis, and confirmed new cases of syphilis in women aged 15–49 years, Denmark, 2010–2024 (n = 963,424 screens)

For pregnancies with confirmed active syphilis infection, Poisson regression adjusting for annual birth counts showed no statistically significant trend over time (IRR = 1.034, 95% CI: 0.998–1.073; p = 0.058), corresponding to an APC of +3.4% (95% CI: −0.9% to +7.2%) indicating that while an increase is suggested, the rate of pregnancies with confirmed active syphilis infection remained stable over the study period. Visual inspection suggested a minor, non-significant peak in 2018 within an otherwise stationary trend (Figure 1).

Initial screening-reactive results showed a median of 109 cases per year (interquartile range: 20.5). Quasi-Poisson regression identified a statistically significant decline over time (β = −0.0224; SE = 0.0056; p < 0.001), corresponding to an IRR of 0.978 (95% CI: 0.967–0.989) and an APC of −2.2% (95% CI: −3.3% to −1.1%), indicating a consistent decline in the proportion of initially reactive screening tests over time.

During the study period, the annual prevalence of syphilis in women aged 15–49 years, reflective of potentially pregnant women, rose significantly. A negative binomial regression demonstrated an IRR of 1.089 (95% CI: 1.057–1.123; p < 0.0001), corresponding to an APC of 8.9% (95% CI: 5.7–12.3). Visual inspection of the graph suggested the steepest rise in recent years (Figure 1).

### Pregnancies with active syphilis infection as a function of self-perceived ethnicity

Of the 172 pregnancies with confirmed active syphilis infection, 83 (48.3%) occurred among women of non-Danish origin. The relative risk among women of non-Danish origin was 2.5 (95% CI: 2.1–2.9; p < 0.0001, exact binomial test), indicating a significant overrepresentation compared with women of Danish origin, and possibly that the subset of pregnant women with pregnancies with confirmed active syphilis infection differs somewhat in ethnic and socioeconomic background.

### Overall incidence of syphilis in Denmark

To evaluate overall national syphilis incidence, we applied a negative binomial regression model to annual case counts for all ages and sexes. The model showed a significant upward trend (IRR = 1.056; 95% CI: 1.035–1.078; p < 0.0001), corresponding to a mean annual increase of 5.6%.

In a combined negative binomial regression including sex and year, both sexes showed significant annual increases in case numbers (women: IRR ca 1.09; men: IRR ca 1.05). The interaction term suggested a steeper increase among women, although the difference did not reach statistical significance (interaction p = 0.059).

**Figure 2:**
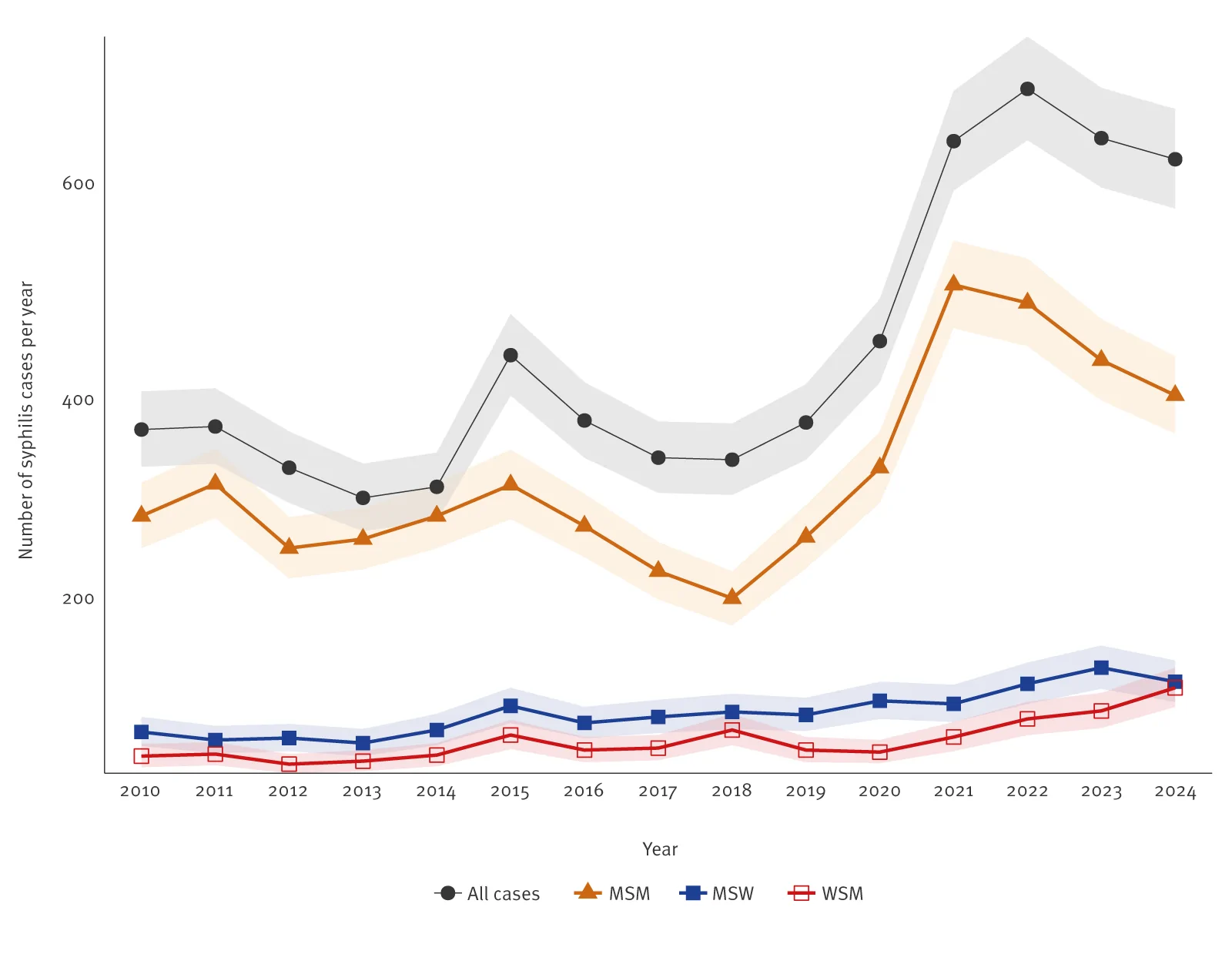
Annual incidents case counts of syphilis, by transmission group, Denmark, 2010–2024 (n = 6,515)

MSM: men, who have sex with men; MSW: men, who have sex with women; WSM: women, who have sex with men.

As expected, men had substantially higher case counts than women. Adjusting for year, the incidence rate ratio for men vs women was 8.57 (95% CI: 7.27–10.10; p < 0.0001), indicating that men had more than an eightfold higher number of cases throughout the study period. Prevalence rates among women aged 15–49 years increased significantly (IRR = 1.089; 95% CI: 1.057–1.122), corresponding to an APC of +8.8% (95% CI: 5.7–12.2). Annual syphilis incidents case counts per sex and transmission groups are presented in Figure 2.

## Discussion

In this nationwide study, we found congenital syphilis to be eliminated, with no case since 2016 and only five cases recorded over 15 years (2010–2024), equal to an incidence of 0.6 per 100,000. According to the WHO, elimination as a public health problem is defined as reducing incidence to a level where the disease burden is negligible, specified for global congenital syphilis as ≤ 50 cases per 100,000 live births [16]. For the European Region, the target for congenital syphilis was ≤ 10 cases per 100,000 live births in 2025, and ≤ 1 case per 100.000 live births in 2030 [17], which is already well achieved in Denmark. Accordingly, Denmark was officially recognised in February 2026 as having eliminated congenital syphilis [18].

Over the 15-year study period, just five cases were identified. Two cases occurred among women arriving to Denmark in late pregnancy who were therefore not screened in time, and two were attributed to maternal infection acquired late in gestation after a negative screening result. Importantly, none of these cases reflected failure of the first-trimester screening algorithm itself. One case was preventable and resulted from a reporting irregularity that delayed initiation of treatment. Following this incident, communication protocols between laboratories and general practitioners were thoroughly revised to prevent similar events.

The stable trend in the prevalence of pregnancies with confirmed active syphilis infection indicates that, unlike several other high-income countries, there has been no resurgence of infection among pregnant women, which indicates a well-functioning healthcare system providing diagnosis and treatment before pregnancy. Despite increasing syphilis notifications in the general population, including among women aged 15–49 years, we observed no temporal increase in congenital syphilis. Together with the stable rate of confirmed maternal syphilis during pregnancy, this provides epidemiological reassurance that the Danish antenatal screening programme continues to support timely diagnosis and treatment, thereby contributing to the continued rarity of vertical transmission. However, ongoing vigilance is needed, as the occurrence of even a single congenital syphilis infection constitutes a sentinel event pointing to potential missed opportunities in testing or treatment [5,19].

The stability of pregnancies with confirmed active syphilis infection is consistent with surveillance reports from other Nordic countries, where antenatal screening coverage is also high [8]. In contrast, several European countries have reported increases in maternal infections since 2015 [8]. A key factor may be differences in the epidemiological dynamics of syphilis transmission, particularly among men who have sex with men and migrant populations, and how these intersect with women of reproductive age [20].

The overrepresentation of women of non-Danish origin among pregnancies with confirmed active syphilis infection suggests that differences in migration history, healthcare access or early antenatal engagement may influence the timeliness of screening. In this context, it is also noteworthy that two of the five recorded cases of congenital syphilis occurred in children born to women arriving in Denmark shortly before giving birth, and the three additional cases were found in children born outside Denmark.

This study’s major strengths include its nationwide design providing an unbiased representation of the entire birth population, linkage of high-quality population and laboratory registers, and mandatory reporting to the national surveillance system, which together ensure near-complete ascertainment of maternal and congenital syphilis. The ability to qualitatively validate each congenital case further strengthens classification accuracy.

However, limitations should be noted. Firstly, the very small number of congenital infections preclude detailed stratified analyses. Secondly, some misclassification of maternal syphilis stage or timing of infection cannot be excluded. Thirdly, because our analyses included only live births, congenital syphilis may be underestimated if fetal deaths or stillbirth caused by untreated maternal infection were not registered as syphilis-related; the authors are aware of one such potential case [21]. Finally, behavioural and demographic data were not available, limiting assessment of individual risk factors or antenatal engagement patterns. It is therefore possible that women with confirmed syphilis differed demographically from the overall screened population in ways not captured by our data. 

Continued surveillance of both maternal and congenital syphilis remains essential. There is a relative lack of knowledge of the obstetric consequences of having a pregnancy with confirmed active syphilis infection, and future studies could explore obstetric characteristics of the group of pregnant women with an initial positive screening result. Integration of microbiological data with antenatal care records could further improve timeliness of case detection and enhance response capacity. Detailed analyses on individual level of the socioeconomic conditions of the group of women with pregnancies with confirmed active syphilis infection may also reveal data leading to future improved surveillance. Finally, comparative analyses across Nordic countries could also help identify programme characteristics that maintain elimination and offer guidance for other European settings.

## Conclusion

Congenital syphilis is eliminated in Denmark, and maternal syphilis prevalence has remained stable from 2010 to 2024 despite an overall increase in syphilis in women aged 15–49 years. These findings indicate that Denmark continues to meet the WHO’s criteria for congenital syphilis elimination. These results highlight the public health value of maintaining high screening coverage and robust surveillance, even in low-incidence countries. Continued attention to vulnerable groups and early antenatal engagement will be essential to sustain these achievements.

## Data Availability

Aggregated data are presented in the article. The individual-level dataset analysed in this study cannot be shared publicly due to data restrictions prohibiting transfer outside the custodial institution.
